# Managing Pervasive Sensing Campaigns via an Experimentation-as-a-Service Platform for Smart Cities

**DOI:** 10.3390/s18072125

**Published:** 2018-07-02

**Authors:** Dimitrios Amaxilatis, Georgios Mylonas, Luis Diez, Evangelos Theodoridis, Verónica Gutiérrez, Luis Muñoz

**Affiliations:** 1 Computer Technology Institute & Press, “Diophantus”, 26504 Patras, Greece; amaxilat@cti.gr (D.A.); mylonasg@cti.gr (G.M.); 2 Engineering Communications Department, University of Cantabria, 39005 Santander, Spain; luis@tlmat.unican.es; 3 Dynamo Ltd., London W6 9DL, UK; evangelos.theodoridis@gmail.com; 4 Telefónica, 39003 Santander, Spain; veronica.gutierrezpolidura@telefonica.com

**Keywords:** IoT, crowdsensing, smart city, campaign, experimentation management

## Abstract

The adoption of technologies like the IoT in urban environments, together with the intensive use of smartphones, is driving transformation towards smart cities. Under this perspective, Experimentation-as-a-Service within OrganiCity aims to create an experimental facility with technologies, services, and applications that simplify innovation within urban ecosystems. We discuss here tools that facilitate experimentation, implementing ways to organize, execute, and administer experimentation campaigns in a smart city context. We discuss the benefits of our framework, presenting some preliminary results. This is the first time such tools are paired with large-scale smart city infrastructures, enabling both city-scale experimentation and cross-site experimentation.

## 1. Introduction

The proliferation of the smart city concept in many science fields, together with the adoption of the Internet of Things (IoT) technologies and the transformation of urban infrastructures, has turned smart cities into a very active research topic. Moreover, smart cities are urban innovation ecosystems where new business models are developed, and other opportunities are emerging based on the continuous activity carried out by active stakeholders when conceiving and developing new urban services and applications. As such, many stakeholders are collaborating within the same environment, either from the public sector, like municipalities, or from the private sector, like companies or small and medium-sized enterprises (SMEs) trying to leverage technological developments. In the same way, governments also aim to provide solutions for the challenges present in modern cities, creating opportunities for improving the quality of life of their citizens.

Although a number of research facilities and smart city experimentation testbeds have been set up all over the world in the last years, the concept of enabling fine-grained experimentation and, at the same time, actively promoting end-user participation and engagement, has not been addressed in a wide manner. In this context, the use of IoT devices together with smartphone applications is a key enabler for the development and consolidation of the innovation ecosystems within the cities, as it allows the validation and further adoption of new services by citizens. People are quite familiar with using such technologies, smartphones as mobile devices can reach corners of the cities where static infrastructure is not available. They also carry and use their smartphones continuously; this presents a key opportunity for researchers to acquire complex and intelligent observations of the urban environment. Additionally, smartphones utilize various integrated sensors, while also having the necessary networking interfaces to communicate with IoT devices like Arduino, smart watches or fitness trackers (i.e., Bluetooth or NFC). On top of that, there is the maker movement, expressed through communities like hackerspaces and Fab Labs, arising in many cities around the world and activating citizens that cooperate with existing activist, and citizen groups in large metropolitan environments.

We present our solution for a more advanced way of conducting experiments within the Experimentation as a Service (EaaS) facility developed in the OrganiCity [1] project. We aimed to provide an unforeseen level of control over how experiments are defined, deployed, and operated in a large-scale smart city platform. In other words, we augment the power of the facility as an ecosystem, by offering new tools to developers, entrepreneurs, and other active stakeholders, which will simplify the way they can conduct experiments and research within the smart cities. Moreover, it will aid them to monitor the experiment execution and progress in real time, giving them the opportunity to verify their design, and helping them with the early identification of weaknesses in the progress. Our discussion here focuses on two tools, the Experimenter Portal and the Smartphone Experimentation Tool. The former is a tool used by all OrganiCity experimenters and acts as the entry point to the facility. The latter solely focuses on crowd-sensing experimentation for Android smartphones, thus providing tailored functionalities. Hereinafter we present their design, implementation, and functionalities, along with a set of preliminary results and experiences collected by experiments conducted by the consortium of the project and other external groups.

Regarding the structure of this article, we first present the state of the art regarding experimentation facilities over smart cities, pointing out how OrganiCity platform differs from the others. Afterwards, we discuss the basic principles of the OrganiCity framework, pairing smartphones with IoT to create virtual urban infrastructures. Then, we follow with the overall architecture and basic concept of the proposed campaign experimentation framework, along with implementation details of the experimental workflow. We discuss indicative experimentation scenarios on top of our platform and present preliminary results and insights from experiments. Finally, we conclude the paper enumerating future work and directions for further exploration.

## 2. Related Work

While IoT experimentation inside smart cities has attracted the interest of a significant number of researchers, nevertheless it still remains challenging to manage sensing campaigns in a systematic, coherent way for all sorts of teams (i.e., experts or non-experts). At the same time, there is a growing trend to combine large experimentation testbeds with data from mobile and IoT devices, which has also not yet been adequately addressed in an integrated way by any large-scale system. We see our work in OrganiCity as an attempt to fill in this existing gap in terms of deployment and management capabilities of such a heterogeneous city-scale system. The experience from recent research shows that smart city experimentation is moving towards using both static and mobile infrastructure, by teams with various backgrounds, which need a way to centrally manage experimentation campaigns.

In terms of experimentation testbeds, the SmartSantander project [2,3] probably built the largest single city-scale IoT research facility, pioneering the experimentation of novel smart city architectures, services, and applications in real-world urban environments. Its emphasis was on managing experiments at IoT device level, focusing on device reprogramming, networking algorithms and routing information across a mesh of devices, and at the same time, permitting data-acquisition tasks, so facilitating the development of urban services on top of the captured data flows. Although experimentation on top of smartphones was implemented, it was a limited approach. Moreover, SmartSantander built interfaces with the FIWARE [4] and FI-Lab ecosystem [5], in order to support interconnectivity with the IoT/Future Internet experimentation community. The Fed4Fire project [6] focused on the development of an infrastructure that can remove all the hassle that is required to access IoT testbeds and use them to develop software that can extend the boundaries of technological domains. Such a strategy is more oriented to the IoT testbed manager side, while OrganiCity focuses more on the usability of the IoT and smart-city data for the development of applications oriented to end-users and citizens.

The smartphone experimentation substrate utilized in OrganiCity builds upon the work described in Reference [7]. The area of crowdsensing in urban areas is discussed extensively in Reference [8], where authors provide both the theoretical background and a review of a number of approaches currently utilized. Our work has similarities with the approaches and ideas described in [9,10,11]. However, apart from aiming at providing a pragmatic solution to the crowdsensing problem, it is also integrated with a smart city platform, allowing end-users to benefit from this interoperability in various ways (e.g., data storage, visualization, interfacing to other systems, community management, knowledge extraction, and urban service creation), and not just basic management of crowd-sensing activities. Our crowdsensing component, as briefly discussed in this work, also allows for a broad set of opportunities for integration of smartphones in the smart city experimentation context. It is essentially a generic tool to allow the use of such devices to produce experimental data as an extension of an existing IoT infrastructure. We also allow easy interconnection to other IoT devices for scenarios like the one in Reference [12], which adds further possibilities to the system.

The mobile crowdsensing paradigm, as well as the associated features and challenges, are further discussed in Reference [13], while in References [14,15,16] incentives and crowdsensing task assignment are discussed in detail. Another recent discussion regarding incentives and mobile crowdsensing is included in Reference [17]. A discussion on the characteristics of people-centric applications and the related challenges and technologies is provided in Reference [18]. The work presented here does not utilize incentivization mechanisms. However, our design allows for such future extensions, while we also employ some very basic related feedback mechanisms. In Reference [19], the various aspects of citizen science are discussed, while References [20,21,22] provide a discussion related to the research questions we are trying to answer in our work as well. Especially Reference [22] aims to provide a system similar in many aspects to our work, with respect to our crowdsensing and smartphone tool. However, we provide a broader tool in terms of functionality. In fact, parts of the services discussed in that work can be replaced by existing functionality in OrganiCity, while our smartphone tool could be extended to directly support the hardware developed for the said system.

Festival [23,24] is another example of federation of existing experimentation testbeds from Europe and Japan in order to provide a unified infrastructure to the research community. However, its focus is not exclusively on the smart city domain, nor does it offer a set of tools similar to the one described here. The CPaaS project [25,26] is an example of another currently ongoing project that has similar goals to OrganiCity. Additionally, there are projects like IoT-Lab [27,28], investigating crowdsourcing and IoT services for supporting multidisciplinary research tasks. Their approach differs, since their services are not tightly coupled with a smart city testbed. Synchronicity [29] is another related project featuring open calls for experimentation, placing a much larger focus on open data markets in the context of a smart city, and not in managing experimentation as a service. In terms of commercially available platforms, Thingspeak [30] is an online platform that, to a certain extent, offers some analytics and other capabilities for managing IoT experiments, but it does not provide the management and monitoring capabilities described in this work. In the US, projects like the Array of Things [31] utilized a smaller-scale deployment with limited management capabilities, while focusing on student communities and integrating IoT aspects as part of educational programmes.

Other existing work focuses on the selection of volunteers for crowdsensing, or on ensuring privacy aspects of the pervasive sensing/crowdsensing components [32,33,34]. While we have not focused on privacy-related aspects in our smartphone tool, we utilize a simple approach based on the transparency of the crowdsensing procedure and data exchange, along with a set of mechanisms to ensure at a basic level that volunteers will not participate in activities which may involve producing data that they think are sensitive.

Regarding our own previous work, we have built our smartphone software components initially over the work of OSGi [35] and Ambient Dynamix [36], adding many layers of functionality for OrganiCity and smartphone IoT experimentation, sensor plugins, automation for software update scheduling, deployment and execution, and social networking, among other features. An early version of the smartphone component discussed here was presented in Reference [7]. A more general discussion regarding OrganiCity is contained in Reference [37], describing in detail its architecture and overall software stack. Furthermore, the potential co-creation capabilities were showcased in Reference [38] by means of a real use-case built on top of OrganiCity. In this work, we focus on the experimentation management aspects and the crowdsensing services provided by OrganiCity.

## 3. The OrganiCity Facility

**Figure 1:**
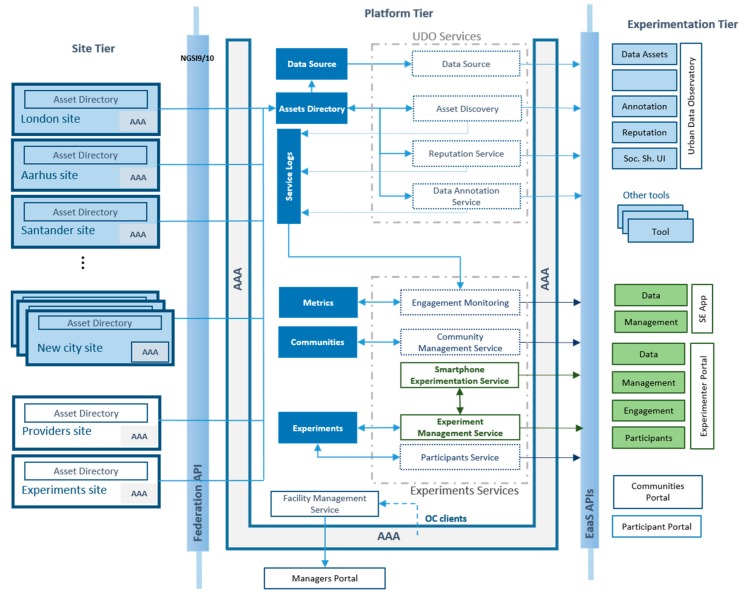
High-level architecture of OrganiCity and relation with the OrganiCity (OC) experimentation management framework.

OrganiCity aims to bring people together at the center of the development of future cities, creating a research facility that federates data and resources from different cities, and that brings about tools to define and develop new-brand urban services. Co-creation with citizens is the fundamental principle of the facility, which is the process of defining novel scenarios for more people-centric applications inside smart cities, by exploiting the use of IoT technologies, heterogeneous data sources, and using enablers that facilitate urban service creation and applications development. It places organicitizens (i.e., citizens actively involved in the project) at the heart of the facility, both by allowing them to shape application use-cases, and by simplifying the ways they can contribute with data and other resources. As its main infrastructure, the OrganiCity platform federates existing smart city facilities (see Figure 1), integrating heterogeneous urban data streams and services. Overall, such federated resources are available to experimenters through an Experimentation as a Service (EaaS) framework.

The OrganiCity platform follows a three tier design, depicted in Figure 1. The first tier, so called *Site Tier*, embraces the federated cities, or sites, that bring their data to the platform through the federation API. Then the Platform Tier provides all the platform services and components to support data, experiments, and platform management. It is worth noting that, apart from a few base platform components, most of them have been developed following a micro service approach so that they run independently and communicate using RESTful APIs. As can be seen in Figure 1, we group the services in Urban Data Observatory (UDO), and Experiments ones. The first group is responsible for data management, while the second one carries out all the task related to experiments. Finally, an additional service performs platform management tasks.

While some of the services residing in the Platform Tier are not public, a subset of them are exposed through EaaS APIs to the OrganiCity tools and instantiated experiments. In a nutshell, UDO services are used by the UDO user interface, while the Experiments ones are mostly integrated within the Experimenter Portal. In addition, other OrganiCity tools make also use of the EaaS APIs. It is also worth highlighting that all services and components make use a common Authentication, Authorization, and Accounting (AAA) system.

The Experimentation Management (EM) service is responsible for maintaining the list of all active experiments in the platform together with the associated data and services, and the attached applications/services of the experiment. While the EM service offers a general purpose management functionality, the Smartphone Experimentation (SE) service provides a tailored service that governs the distribution of the experimenters to the participants’ Android phones. As can be observed in Figure 1, both EM and SE communicate in order to perform the orchestration and management of the experiments in the platform associated with both IoT and smartphone devices. Apart from the corresponding APIs, both EM and SE services are exposed through the Experimenter Portal web interface and an Android application, respectively.

Following the dominant concept in IoT experimentation, for each experiment a virtual testbed is created by associating the experiment instance to the following platform entities:

- Experiment team: list of OrganiCity users allowed to manage the experiment.
- Data assets: each experiment is attached to the data created by its applications. In addition, the experiment data can be made public if desired.
- Participants: list of users who decide to take part in the experiment as users of its applications.
- Experiment scope: temporal and spatial limits of the experiment.

As can be observed, from the platform perspective, experiments are not associated with any city, so that it is possible to create cross-city experiments. Finally, it is worth indicating that experiments are given AAA credentials, so that they are seen as entities whose access rights can be managed (i.e., similar to applications or users).

Although it is possible to experiment with different IoT technologies, data, and applications, this work mainly focuses on crowdsensing scenarios, where people-centric smart city applications gather data under real conditions. Our tools help guiding participants to achieve the quantitative and qualitative objectives of the experimenters. In this context, smartphones are essential parts of the picture, as they contribute data to the OrganiCity with their own integrated sensor readings, or wirelessly communicate with other IoT devices, such as Arduino boards, acting as data mules.

As regards the federation with additional cities, they would need to deploy their own OC instance, encapsulating their urban services pertaining to such a case under a common administration (e.g., a city sharing assets from IoT deployments, a citizen contributing data from IoT devices, an experimenter creating assets during an experiment). Depending on the cities, they may include heterogeneous data assets, such as IoT devices deployed in the cities, smartphones and/or datasets from open data platforms. To this end, a Federation API allows cities and users to integrate such data assets within OC.

Publicly running since October 2016, the OrganiCity facility allows the development of novel services, applications, and real-life testing of new technologies within the smart city. To this end, it supports different types of experiments, being the most common cases the following:

- Urban data collection: experiments involve engagement of users with their smartphones, facilitating data collection from static IoT devices, which are not directly connected to the Internet.
- Smartphones experimentation: these experiments involve deployment and execution of code on smartphones carried by the participants, producing data from the integrated sensors/interfaces, or acting as proxies to other IoT devices.
- Knowledge co-creation: experiments involve extraction of knowledge, by creating annotations, evaluations of the observation provided by the platform, event detection, etc., by the participants.

Specifically, for smartphone experimentation, the overall idea is that citizens participate in the above experimentation cases as participants transparently during their daily life. Experimenters essentially utilize the data generated by static or mobile IoT devices, as well as sensors and networking interfaces of the smartphones carried by volunteers; such data is fed to the SE component, and forwarded to the OrganiCity facility as new data assets, being the whole process transparent to the experimenters. On the volunteer’s side, incentive schemes and rewarding mechanisms are used to keep citizens motivated to participate in the crowdsensing activities.

Finally, with respect to security and privacy, we should again mention that OrganiCity utilizes a common AAA framework throughout the system, having mechanisms in place to guarantee the security of data produced within the project, while also taking into account the European and national regulations and directives that were in place during the project’s lifetime. As also mentioned in the next section, for the part of mobile end-users, we have actively sought to have them anonymous and keep as little information about them as possible, while also giving them ways to directly monitor the kind of data that are being produced by their devices and uploaded to OrganiCity. 

## 4. Experimentation Management

One of the main targets of the OrganiCity facility is to provide the means to perform agile and systematic experimentation for a wide set of diverse experiments, coming from different application fields. On the other hand, as experimentation in cities embraces multiple actors, the entry barrier to start experimentation must stay as low as possible. Bearing this in mind, OrganiCity provides an experiment management (EM) component that brings about a simple interface to setup and manage experiments, so that the underlying complexity is transparent to users. The EM component is split into two main parts: a backend (REST API) that interacts with the rest of the facility; and a front end, the so-called Experimenter Portal, which provides the experimentation functionalities to the users in a friendly way. To our best knowledge, the OrganiCity EM service is the first one of its type devoted to the creation and management of experiments in such a large scale inside a smart-city ecosystem.

As mentioned before, the EM component provides the means to handle experiments from different nature. In addition, the OrganiCity facility also provides a set of advanced tools to, on top of the EM, simplify experimentation in some areas. Amongst those fields, crowdsensing by using smartphones is of utter importance and has been natively supported in OrganiCity by means of the smartphone experimentation (SE) tool. In the following, we will introduce the main concepts of experimentation in OrganiCity, in order to explain better the use of the SE tool.

### 4.1. Experimenter Portal

The Experimenter Portal essentially offers a graphical interface to exploit the functionalities brought about by the EM component. A user registered into OrganiCity, and granted experimenter role, can access the portal and create experiments that, to some extent, can be seen as management units to interact with the rest of the facility. Among other functionalities, the portal offers the experimenter the ability to access a set of application-specific tools and enablers offered by the OrganiCity facility, documentation and support channels besides the options to create, configure and execute experiments. Additionally, experimenters can select data from the cities or the experiment, called data assets, to be consumed in an experiment or examined, in order to process them and carry out further developments or analysis. Data assets generated within the scope of a particular experiment can be presented on both maps and lists. Finally, through the EP, experimenters are able to configure and manage applications that are part of the experiment, and to define experiment metrics that can be monitored in real time.

For the configuration and integral management of the experiment during its entire lifecycle, the Experimenter Portal enables experimenters to control parameters of the execution of the experiment both before and during its deployment. This refers to their ability to define spatiotemporal requirements for the data collection including restrictions on:

- Where the experiments are executed by defining multiple geographical areas of interest, in the form of polygons.
- When the experiments are executed by defining discrete times of interest (e.g., there is little interest in data collection between 2–6 AM) for each polygon previously defined.
- The minimum or maximum number of data points within each polygon. For example, if measurements in an area have reached a critical mass, the system can coordinate participants to other areas to achieve better coverage.

The aforementioned restrictions help to achieve a more focused and resource-friendly execution of the experiments; not only is the geographical area of execution important, but the temporal dimension is also taken into account. Apart from the ability to define these restrictions, the system provides feedback options and interfaces to monitor the progress and current state of the execution of the experiments and the degree of fulfillment during the execution. For instance, the percentage of the requested measurements already collected.

Besides that, the Experimenter Portal offers advanced functionalities that allow experimenters to develop more complex experiments. In this last case, through the Experimenter Portal, the experimenter is able to configure a smartphone experimentation application, selecting a set of plugins that can be used within the scope of the experiment. Further configuration and management of the experiment is carried out by means of the smartphone experimentation tool that is presented in the next section.

### 4.2. Smartphone Experimentation Tool

In the particular case of experimentation with smartphones, the experimentation component permits to distribute crowdsensing tasks and execute them from a smartphone application installed on the participants’ phones. Three main components are responsible for carrying out this type of experiments: the Smartphone Experimentation (SE) service, the Smartphone Experimentation application, and the Sensor and Experiment plugins.

Implemented using the Spring Boot application framework [39], the SE aims to orchestrate the operation of the smartphone applications within a particular experiment. It acts as a repository, allowing the distribution of OSGi [35] plugins created and uploaded by experimenters, as well as a data collection hub for the measurements generated from participants’ phones. The OSGi framework used for the development of the sensing plugins is used to allow the update of Android application’s functionality dynamically, without the need for a new version from the Google Play Store. In addition, it provides a degree of transparency as the end-users can select which particular plugins they wish to install on their phones.

The Smartphone Experimentation application is a mobile application for Android devices that acts as an execution wrapper for the OSGi plugins selected by the volunteers of the experiment. Volunteers can participate in different experiments from a single application, simplifying the process and making it more familiar. The execution of such experiments within the application is dependent on participants’ preferences (i.e., in terms of sensing modalities that they wish to contribute), thus greatly simplifying the deployment, bookkeeping, and maintenance for both experimenters and participants, while also allowing for better control over the actual code executed and their personal data potentially exposed to OrganiCity. This essentially means that since smartphone users choose what kind of data and sensors they wish to share, only those experiments that utilize this set or subset of plugins that correspond to the user preferences can be executed on their devices. Furthermore, such experiments additionally have to be approved for execution by the smartphone users before actually starting to collect data. Moreover, end-users can actually check the content of the messages exchanged with OrganiCity, through a dedicated tab in our app. Therefore, the users are both aware and in direct control of a number of basic aspects related to their privacy.

Additionally, the SE service also calculates several statistics about the execution of the experiment, providing progress insights to experimenters. Using Push Notifications [40], the SE service is also capable of delivering notifications to participants in two forms:

1. Participants are notified of their achievements while contributing to an experiment.
2. Experimenters can also send notifications to participants with updates on experiments’ results.

To develop a new sensor or experiment plugin, the experimenters need to implement their business logic according to specific guidelines, and then submit it to the smartphone service to start distributing it among OrganiCity volunteers. Sensor plugins comprise the low-level functionality with respect to producing data, while Experiment plugins bundle the sensor plugins in the context of an experiment. The development of a simple plugin for an experiment requires 30–100 lines of Android code. Once the plugin is inspected and validated by the OrganiCity consortium, the smartphone service permits its distribution to OC volunteers, who both run the application and participate in a specific experiment. The selection and execution of the distributed plugins from the smartphone application start the process of feeding data to the smartphone service, which forwards such information to OrganiCity, in an anonymized format. From the application, participants have the ability to stop or pause the execution of the experiment, or even they can decide to leave the entire process.

Regarding data types and measurements supported, a great deal of flexibility is provided to the developers: they can either opt for the readily available sensing plugins, which support the most common sensors found in Android smartphones (e.g., GPS, microphone, WiFi, Bluetooth, barometer, etc.) or they can develop their own plugins to handle the specifics of their own experiment. With this in mind, the format and data type of the results gathered is mostly up to the experimenter; as a tool, the management platform is agnostic about the nature of the measurements. The crucial parameters in the tool refer to the spatiotemporal and other types of restrictions defined for the experiment, and these are the ones of use to the execution of the experiments. For instance, the tool is agnostic of whether the measurements gathered are temperature or humidity values. The interpretation and visualization of the experiments’ results as meaningful entities is up to the IoT experimenters. The tool visualizes the progress of the experiment execution, (e.g., how many measurements were produced over the map and over a certain period). Such results can be downloaded during or after the execution of the experiment in CSV or JSON format.

Finally, it is important to note that in smart city environments certain privacy and security issues for the participants’ personal data and their daily activities arise. To interact with OrganiCity, experimenters need to use OAuth 2.0 authentication. OrganiCity services and third-party applications of the experiments are considered as micro-services, authenticating the users with simple but well-defined authorization flows, using a pair of authentication keys (private and public) as well as the user (bearer) tokens that identify users across all services. On the privacy level, user anonymization methodologies [37] are enforced, including generalization and suppression. Generalization is the process of providing the recorded data based on their category and not their actual values, while suppression is the process of removing data that can identify users for the final experiment results provided to experimenters. In our case, to generalize the data gathered during experimentation, it is provided to the experimenters with a coerce location of the measurements, instead of the exact GPS coordinates provided by the smartphones. Apart from that, in the case of the smartphone service, it merges the data collected in the same region with data collected by other participants. Last, but not least, the results of the experiment are aggregating data in regions, according to the experimenter preferences. For example, we group them based on the low coverage by a few participants, or by a specific time of the day.

## 5. Experimental Validation and Results

In order to validate the Experimentation-as-a-Service model defined in the core of OrganiCity, multiple crowdsensing campaigns were deployed and managed in three European cities (Patras, London, and Santander) to highlight the possibilities and deficiencies of the experimentation management. To this end, volunteers from the three cities were recruited to participate in multiple runs of the following campaigns:

- WiFi campaign: the target of the experiment was to record WiFi access points in London, Santander, and Patras (similar to Reference [14]), and to use the data gathered to provide geolocation information to citizens.
- Noise campaign: the goal was to record noise levels using the microphones of smartphones in the city centers of the same locations.
- Air quality campaign: the goal was to collect readings from a portable air quality monitoring station (based on Arduino), which is paired with a smartphone collecting the data periodically over BluetoothLE.

### 5.1. Configuration and Setup of the Experiment

The overall process to configure a crowdsensing experiment is described in Table 1. The first step to start the experimentation within the OrganiCity facility is the registration of an experiment. As mentioned above, an experiment constitutes the central management entity for OrganiCity, which embraces a spatiotemporal scope and the applications to be run on it. In this section, we will present the more relevant phases to configure and execute a WiFi campaign experiment.

**Figure 2:**
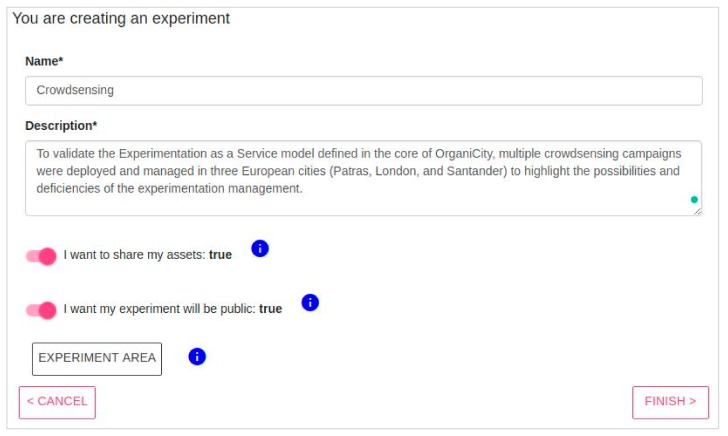
Screenshot of the Experimenter Portal to provide general experiment information.

*Step 1*: Definition of generic information (see Figure 2). Once the experiment concept is defined and the regarded application properly tested, the experiment can proceed with the experiment registration in OrganiCity. In this sense, it is required that the experiment provides essential information regarding the experiment such as its name and description as depicted in Figure 2. Along with the name and description, the experimenter is also required to provide the overall duration of the experiment and the privacy level of the assets generated by the experiment. The privacy level defines whether the information generated by the experiment may be used by other experimenters.

**Figure 3:**
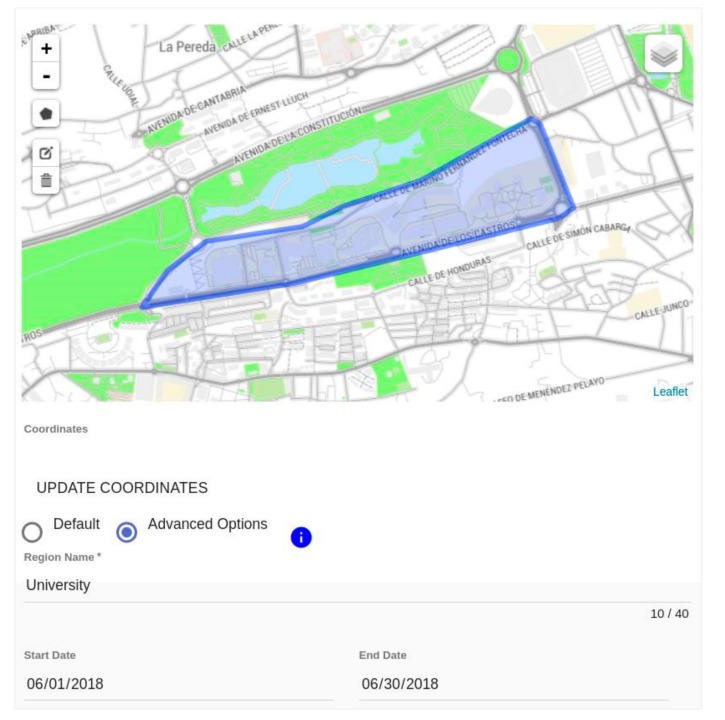
Screenshot of the Experimenter Portal to exemplify the definition of the spatiotemporal experiment constraints.

*Step 2*: Definition of the spatial experimentation constraints (see Figure 3). Once the basic definition of the experiment is provided, more fine-grained configuration of the experiment can be set within the experiment area. The experiment area is made of a set of regions built as polygons, each with a different name, as shown in Figure 3. Furthermore, the experimenter can define different time limits of each region, provided those limits are within the overall experiment duration. On top of that, the experimenter is also allowed to define the relative importance of one region over the others and to establish score limits for each. This detailed configuration, while generic, is especially appealing for crowdsourcing experiments, in which information from different regions can have uneven importance.

*Step 3*: Application creation. As mentioned above, an experiment has a spatiotemporal scope and embraces several applications. Apart from the application name and description, the experimenter must indicate the type of application and an end-point through which the application can be accessed. It is worth highlighting that an OrganiCity application is defined as an entity that generates or consumes data assets. In this sense, the application binds the OrganiCity experiment to the actual running code by using the proper security procedures, see Reference [38] for detailed information.

**Figure 4:**
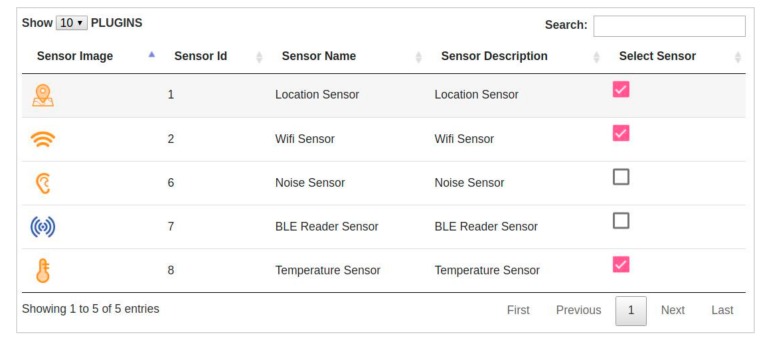
Screenshot of the Experimenter Portal to select experimentation plugins.

*Step 4*: Selection of sensor plugins (see Figure 4). While application definition is rather generic, given the deep integration of the SE tool with the OrganiCity core, the Experimenter Portal brings about additional functionality to configure them. In this regard, as illustrated in Figure 4, experimenters can select the set of plugins for sensors required for the specific experiment. These plugins can be already available or uploaded as new ones if necessary. Furthermore, the experimenter can decide whether its plugins are private or publicly released so that other experimenters can use them. Figure 4, experimenters can select the set of plugins for sensors required for the specific experiment.

*Step 5*: Experiment code upload: As a final step, the experimenter needs to upload the actual experiment code. This piece of code embeds some information related to the experiment so that it can properly interact with OrganiCity, and can be as simple as a procedure that aggregates the data from various sensor plugins, or a more complex process that combines data and generates new measurements. This code is validated by the SE service and then the application is ready to be distributed to the SE app installed on the volunteers’ smartphones.

**Figure 5:**
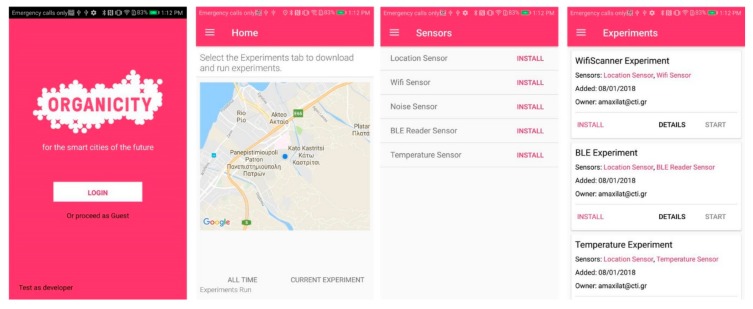
Screenshots from the Smartphone Experimentation Android Application.

*Step 6*: Finally, volunteers can install the smartphone app by downloading it from OrganiCity website. Inside the app, selecting, enabling, and installing sensors and experiments can be easily performed by selecting/deselecting a checkbox. Experiments are downloaded in the background and execution starts automatically. Figure 5 contains screenshots from the SE Android application. The first two screenshots are the lists of sensing plugins available and the experiments. Participants simply need to select via the checkboxes the ones they wish to use and then click the respective “Install” button to start the installation process. The third screenshot is from the home tab of the application when the user can track his location on the map and basic information on the status of the experiment executed. In certain experiments, the current data are also available (if their data types can easily be depicted). This screen contains amongst others information about the total data points gathered thus far, the time spent online, ranking of the user and achievements acquired both under the scope of the experiment and in general. In addition, this tab contains a button to download the participant’s contribution and a couple of graphs showing the contributions of the user during the previous week and the areas visited as a heat map.

Once the experiment is running on smartphones, various statistics from the data generated are available in real-time on the users’ devices, concerning both the current experiment and the overall statistics for a specific volunteer.

On the experimenter’s portal results tab, the experimenter can visualize information about the progress of the experiment. Information such as completeness and coverage (both spatial and temporal) are made available for each area of interest. The data are presented either in summary tables (e.g., for the per-hour completeness) or using on-map visualizations. The map visualizations are available to experimenters in near real-time, aiming to provide the experimenters with a better understanding of the progress of the experiments’ execution. The produced data are available in an aggregated manner, (i.e., no individual device/user can be monitored directly), while our visualization components do not provide live streaming, due to privacy and performance concerns.

Regarding visualization, since this tool aims to be a generic platform serving various types of experiments and data, we mainly visualize aspects such as the progress of the experiment, completion according to the constraints set, etc. The visualization of the specific data according to their datatype for each experiment is left to the experimenters. Upon the completion of an experiment, or even during the execution, experimenters can easily download the data produced in various formats (e.g., JSON, CSV, etc.). Additionally, experimenters can use the data available to better incentivize and engage participants.

**Figure 6:**
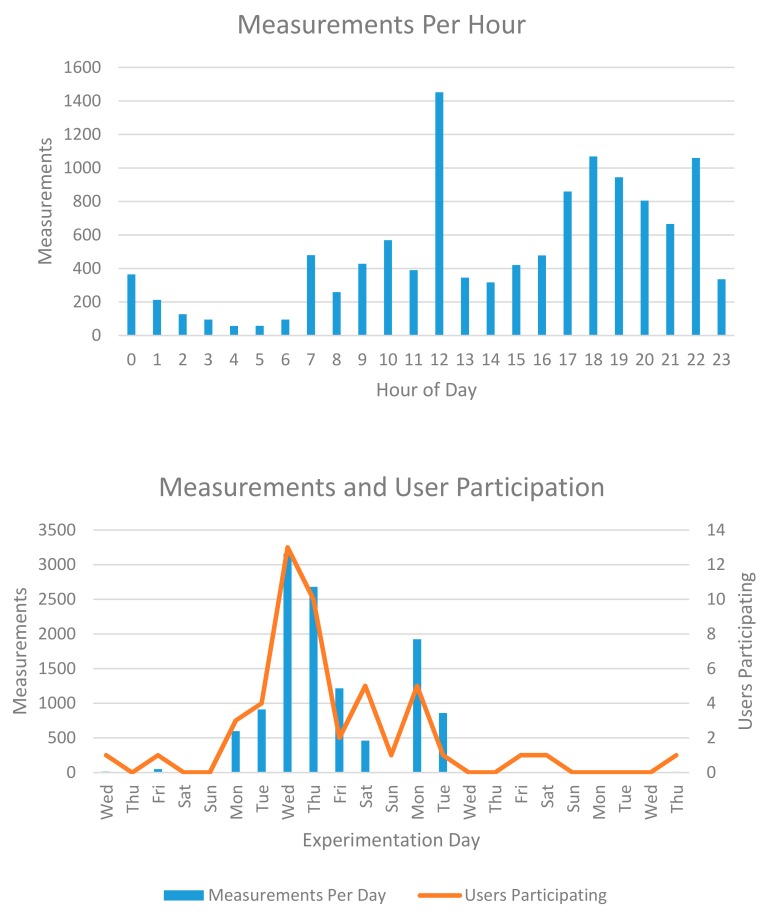
Results from the sensing campaigns performed.

The summarized figures regarding this experiment are displayed in Table 2 and Figure 6. The WiFi experiment was performed over 23 days in three cities, with a total number of 16 participants carrying their smartphones. These participants were mainly members of the research groups involved in OrganiCity, since this set of results serves as a showcase of the system. Although no special incentives policy was utilized to engage end-users, the results produced are generally in line with similar previous attempts (e.g., [14]), in terms of actual measurements gathered and area covered by the users.

As mentioned previously, the system can output information in real-time regarding, for example, the distribution of readings collected for each time of the day, or other metrics for end-user engagement, which we utilized to provide some general directions to the involved participants, mainly regarding areas left with no readings produced. In Figure 6, we can see that the participants, in general, contributed with more readings during the afternoon, and that after an initial growing spike of interest, they stopped contributing after a few days.

### 5.2. Comparison with Other Similar Experiment Workflows

We now attempt to draw a comparison to similar experiments carried out as for example in References [12,14], and how the adoption of our tools would change their experiment workflow. In Reference [12], measurements of air quality in terms of particle concentration were carried out by teams of participants carrying smartphones, while in Reference [38] participants recorded available WiFi networks with their smartphones in certain areas, together with incentive mechanisms. Comparing with the experiments presented in Reference [14], we have similar numbers in terms of people participating in the experiments (13 in our case and 16 in Reference [14]). We also see a higher retention number for users in Reference [14]; however, they also utilized monetary incentives, while we did not utilize any similar incentivization in this case, while achieving similar results for the majority of time.

Another similar example would be Reference [41], which was one of the first examples of works using participatory sensing to produce urban noise maps. In an experiment case similar to the first one, researchers could utilize the provided BluetoothLE plugin and functionality for data relay and storage in OrganiCity, and focus only on the visualization part described in their work. In the second case, again having smartphones sensing the WiFi networks could be easily achieved by using the provided functionality, while letting the OrganiCity app handle the rest. Some tangible benefits in both use-cases would be the following:

- Integration within a smart city ecosystem like OrganiCity, which provided many additional options for data storage, visualization, and APIs to expose the produced data.
- Tools to control and monitor the ways that the volunteer groups handle their task to gather data, without having to, for example, develop a web tool to monitor the progress of the “experiment” and quickly spot areas that have not been covered yet.
- Place less focus on the mobile app development part for Android, and save time utilizing the available software components.

We should also mention that a certain restriction by using our tools would be the limitation to use only the Android platform (due mainly to the utilization of OSGi), which could be limiting to several types of studies.

### 5.3. Exploitation of OrganiCity

By means of open calls [42], OrganiCity aims at drawing the attention of the different stakeholders involved in the experimentation lifecycle, to help the facility to evolve according to the experience gathered from actual experiments. In terms of numbers, 25 experiments run on top of OrganiCity for more than six months. After this first experimentation period, which helped to mature the facility, a second round of 17 experiments are currently exploiting OrganiCity for different purposes involving cities and business companies. Since the second experimentation period has not finished yet, the information reported hereinafter will mostly focus on the outcome of the first 25 experiments. As was commented before, the data stored within OrganiCity comes from both federated cities as well as experimentation, and involves both data assets and historical records from those assets.

**Figure 7:**
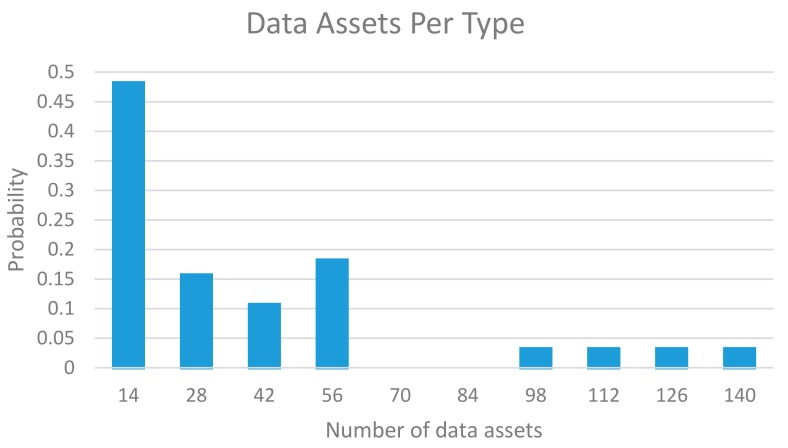
Probability distribution of types of data assets created by experiment.

As a consequence of the experimentation activities carried out during in OrganiCity during 2017, more than 4000 new data assets have been created within OrganiCity. In turn, these assets belong to 58 different types, attending the different application scope of the experiments. In this sense, Figure 7 depicts how the different data assets are distributed among the corresponding types by means of the probability distribution. As can be observed, in most of the cases the number data assets of a particular type are below 60, which indicates the high diversity of the experiments running on the platform. In particular, the results show that around the 45% of the new data types only have 14 data assets or less. On the other hand, only occasionally a new type has more than 100 data assets.

**Figure 8:**
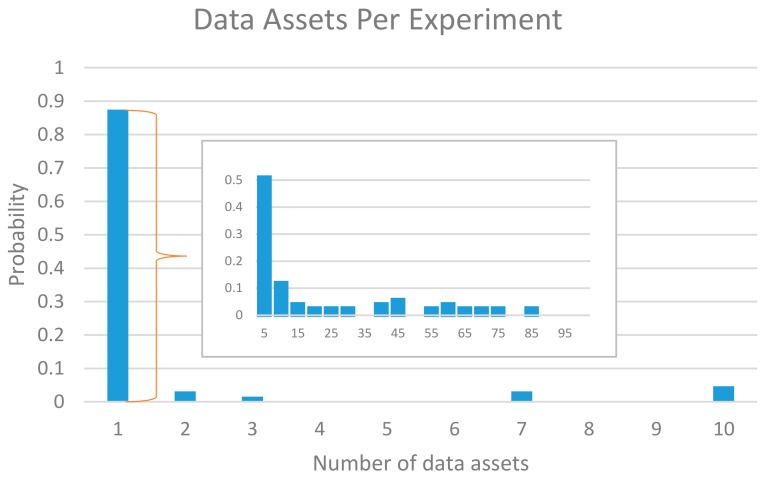
Probability distribution of the number of data assets created by the experiments.

Furthermore, we have observed a rather high variance on the number of data assets created by the experiments. In this regard, Figure 7 illustrates the distribution of a number of data assets created by experiments. As can be observed, most of the experiments generate fewer than 100 new data assets, while in a few cases, they have generated around 1000. It is worth highlighting that, in order to keep control of the facility during the open calls, the number of assets per experiment was bounded to 1000 (i.e., no experiment could create above 1000). In addition, Figure 8 also reveals that almost half of the experiments have only created five or fewer data assets.

As for the assets federated by the cities, currently, OrganiCity offers more than 12,000 data assets which can be exploited by experimentation and correlated with the crowdsensing information provided by the SE tool. This information belongs to a wide range of urban services and IoT sensors, embracing from air quality sensors to parking lots occupancy, and is openly accessible for experimenters through the Urban Data Observatory [43].

Regarding the smartphone-specific tool discussed here, it has been used by three of the experiments yielding approximately 700 new data assets. As can be noted, the rate of information generation of these experiments is quite high compared with the rest of experiments, further highlighting the relevance of this tool. With respect to the management functionality provided by this subsystem and the constraints mentioned previously, from the initial feedback gathered from the first open call, the definition of spatial areas was more appealing to them, in comparison to the temporal ones. This might be the result of their specific experiments not affected by the different time of the day while gathering measurements. An interesting fact was that using integrated incentive mechanisms was not as appealing as a feature; the teams preferred to use their own ones, should there be available mechanisms from OrganiCity. In the case of award mechanisms, developers preferred virtual awards and monetary ones. The teams also used exclusively the already available plugins for functionality, while they also made comments for future additions regarding the ease of identifying specific contributors to their experiments. This suggests that using anonymization techniques in all cases might not suit all experiment scenarios, as we initially had in mind.

Furthermore, once the first open call concluded, OrganiCity has started to record historical data, leveraging the COSMOS [44] ecosystem created under the FIWARE initiative for big-data analysis. It is worth mentioning that the repository of historical data is continuously being fed by both federated cities and experiments, currently running as part of a second experimentation period.

## 6. Conclusions and Future Work

In this article, we presented a solution that facilitates the deployment and management of crowdsensing campaigns over cities federated in the OrganiCity facility. We believe that smart city IoT ecosystems should further encompass crowdsensing aspects, since such a move will result in more useful and pragmatic applications, through the engagement of different end-user communities. In order for this people-centric vision to materialize, we provide a tool to make it easy for people aiming to conduct crowdsensing campaigns to define more precisely what they would like as input from the people participating in such activities, as well as additional feedback that makes it easy to characterize their progress quantitatively and qualitatively. It is important to note that such information can be provided by the system automatically. There is no need for experimenters to reinvent the wheel every time they perform an IoT.

So far, over 25 teams have been utilizing the ecosystem of OrganiCity, which is exactly the end-user target group for the tools described here. The set of metrics and feedback options provided by our tools targets both experimenters and volunteers, making it easier to monitor the progress of the whole procedure in real-time. Experimenters are able to see whether participants’ contributions fulfill the spatiotemporal constraints they have defined, while participants can easily track their own contributions. Thus, the system provides the opportunity for developers to correct the course if they see through the system that their campaign design underperforms. Such information can also be directly fed into incentive mechanisms and gamification components, providing an additional level of control over the progress of the campaigns.

Regarding pros and cons of our approach, in our opinion the standout feature of our system is that it is essentially integrated in a general-purpose framework for urban experimentation, in many cases offering features that were partially present in much smaller-scale ones. It is not a “one-size-fits-all” solution to managing pervasive campaigns, but in many cases it simplifies the overall procedure, allowing for quick prototyping and automation of a number of aspects: collection and storage of data, real-time monitoring and visualization, automatic software updates for mobile devices, automated reports, among others. However, in a number of cases we have seen that experimenters would have preferences that clashed with our design, such as in the case of mobile end-users’ anonymity, which is provided by default in our system. Also, as part of a complex ecosystem like OrganiCity, additional revision will be required to simplify the user interface and streamline the whole experience for the end-users.

In the future, we plan to offer as an option to use a number of additional incentive schemes and rewarding policies within the system, along with the integration of gamification aspects, based on the metrics obtained from the platform. Finally, further use of the tools by third-party experimenter groups will also help to enhance the system’s functionality in more meaningful aspects.

## Figures and Tables

**Table 1:** Overview of the configuration and setup steps for experimentation.

Step	Description
Definition of generic information	Essential information regarding the experiment like name, overall description, time duration, and the privacy level of the data generated by the experiment.
Definition of spatiotemporal experimentation constraints	Fine-grained configuration of the experiment by defining sets of polygon regions, different time limits in each region, relative importance of a single region.
Application creation	Application name and description, type of application, and an end-point through which the application can be accessed.
Selection of sensor plugins	Select the set of sensor plugins required. The plugins can be either available or be uploaded as new. Decide whether plugins are private or public.
Experiment code upload	Upload the actual experiment code.
Install the smartphone app	Volunteers install the smartphone app and then select, enable, and install sensors and experiments.

**Table 2:** Experiment configuration.

Parameter	Value
Cities	3
Participants	16
Regions	8
Experimentation Days	23
Measurements Requested	16,000
Measurements Collected	10,940
Area of Regions Selected	$11\;{\mathrm{Km}}^{2}$
Area Covered by Measurements	$0.86\;{\mathrm{Km}}^{2}$
